# Chromatin Remodeling in the Brain-a *NuRD*evelopmental Odyssey

**DOI:** 10.3390/ijms22094768

**Published:** 2021-04-30

**Authors:** Sarah Larrigan, Sujay Shah, Alex Fernandes, Pierre Mattar

**Affiliations:** 1 Department of Cell and Molecular Medicine, University of Ottawa, Ottawa, ON K1H 8M5, Canada; salarrigan@ohri.ca (S.L.); sujshah@ohri.ca (S.S.); alfernandes@ohri.ca (A.F.); 2 Ottawa Health Research Institute (OHRI), Ottawa, ON K1H 8L6, Canada

**Keywords:** NuRD, neurodevelopment, neurogenesis, gliogenesis, intellectual disability, autism spectrum disorder, chromatin remodeling

## Abstract

During brain development, the genome must be repeatedly reconfigured in order to facilitate neuronal and glial differentiation. A host of chromatin remodeling complexes facilitates this process. At the genetic level, the non-redundancy of these complexes suggests that neurodevelopment may require a lexicon of remodelers with different specificities and activities. Here, we focus on the nucleosome remodeling and deacetylase (NuRD) complex. We review NuRD biochemistry, genetics, and functions in neural progenitors and neurons.

## 1. Introduction

Neurodevelopmental disorders (NDDs) are a highly heterogeneous and common group of conditions. NDDs include a number of “spectrum” conditions that can range widely in their severity, such as autism, attention-deficit/hyperactivity disorder, intellectual disability, epilepsy, and developmental delay. Relatedly, NDD etiology is highly complex. Genetics play a major role in NDDs, with hundreds of genes linked to disease. Moreover, the mutational landscape of NDDs is additionally complicated, with de novo mutations, structural rearrangements, and risk alleles all making important contributions [1,2,3,4]. Adding further complexity, environmental factors such as fetal alcohol exposure and gestational maternal immune activation are also causative.

To understand the mechanisms that drive NDD pathogenesis, we must first understand neurodevelopment. How is the production of different cell types and tissues programmed? Nowhere is this question more challenging to answer than in the developing brain, where hundreds of different neural subtypes must be produced, each of which must be programmed to ‘wire and fire’ with exquisite precision. Since cellular diversification must be achieved via the configuration and reconfiguration of an invariant genome, the role of epigenetic regulation in neurodevelopment has been the subject of intense interest. Indeed, chromatin remodelers—the genes responsible for epigenome dynamics—are prominently associated with NDDs. Deciphering how disparate chromatin remodeling functions contribute to neurodevelopment and NDDs remains an important unsolved problem. Here, we focus on the contribution of the nucleosome remodeling and deacetylase (NuRD) complex to brain development and NDD etiology.

## 2. NuRD Complexes

**Figure 1:**
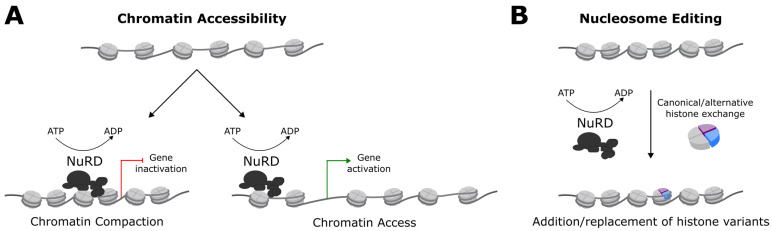
NuRD and nucleosome remodeling. (**A**) The ATPase/Helicase domain of Chd4 consists of two RecA-like lobes surrounding a DNA binding cleft and ATPase active site. This enzymatic domain uses a ‘twist defect’ mechanism to break histone-DNA contacts, resulting in a ~5 bp DNA translocation relative to the nucleosome [21,37]. DNA translocation regulates nucleosome spacing, thereby affecting chromatin access. Nucleosome sliding can lead to compaction or decompaction of the local chromatin environment, which can expose or occlude regulatory elements to alter the expression of target genes. During cerebellar development, NuRD suppresses the accessibility of regulatory elements, and decommissions a subset of promoters [29,38]. (**B**) Nucleosome remodeling can also facilitate the deposition of new histones and histone variants [39]. For a comprehensive view of nucleosome remodeler mechanisms, see [40].

The NuRD complex was first characterized by several groups more than 20 years ago [5,6,7]. Among chromatin remodeling complexes, NuRD uniquely possesses histone deacetylase (HDAC) activity in addition to nucleosome remodeling activity. These key enzymatic activities are provided by (class I) histone deacetylases, typically HDAC1 and HDAC2, as well as Chromodomain Helicase DNA-binding (CHD) proteins, namely CHD3 (Mi-2α), CHD4 (Mi-2β), and CHD5. CHD proteins are members of the SWI/SNF (switching defective/sucrose non-fermenting) superfamily of chromatin remodelers, and contain SNF2 (sucrose nonfermenting 2)-like ATP-dependent helicase domains that mobilize nucleosomes (Figure 1).

**Figure 2:**
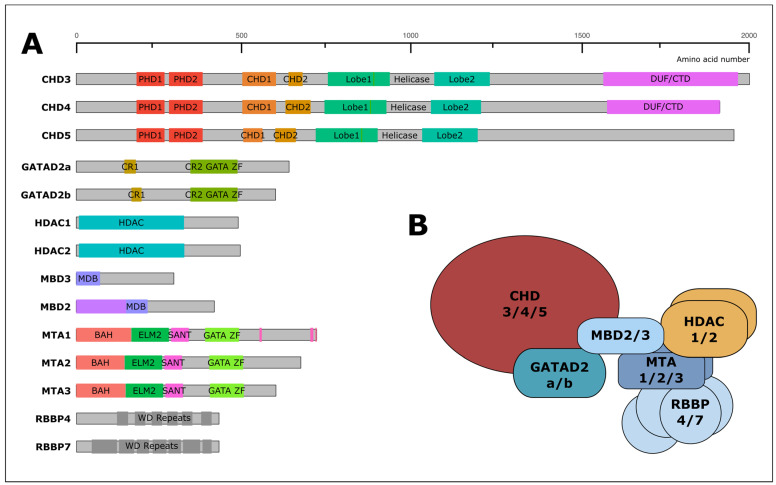
The NuRD complex. (**A**) Six core subunits of the NuRD complex have been identified, with each subunit being encoded by a homologous gene family. These are the chromodomain-helicase DNA-binding proteins (CHD3/CHD4/CHD5), the GATA zinc finger domain proteins (GATAD2B/GATAD2A), the histone deacetylases (HDAC1/HDAC2), the methyl-domain binding proteins (MBD2/MBD3), the metastasis-associated proteins (MTA1/MTA2/MTA3), and the retinoblastoma binding proteins (RBBP4/RBBP7). CHD and HDAC subunits confer both ATP-dependent nucleosome remodeling activity and histone deacetylase activity, respectively. Not all of the subunits are exclusive to the NuRD complex. For instance, RBBP4/7 subunits are also found in Polycomb complexes, as well as the CAF histone chaperone complex. (**B**) Proposed stoichiometry of the complex (see text for details).

The NuRD complex contains a number of additional subunits, each of which is encoded by multiple paralogous genes (Figure 2a). Recent research from multiple groups suggests that a metastasis-associated protein (MTA) 1/2/3 dimer recruits four Retinoblastoma-binding protein 4 (RBBP4; RbAp48) and/or RBBP7 (RbAp46) proteins, along with two HDAC1/2 subunits to form a histone deacetylase module [8,9,10,11] (Figure 2b). Methyl-CpG-binding domain protein 2/3 (MBD2/3) binds across the MTA1 dimer interface and recruits a single GATA zinc finger domain containing 2A (GATAD2A; p66α) or GATAD2B (p66β) protein. GATAD2 proteins in turn interact with a single CHD3, CHD4, or CHD5 subunit. As CHD, GATAD2, and MBD subunits are thought to be monomeric within NuRD, the recruitment of each of these proteins appears to be mutually exclusive with their respective paralogs [12,13]. The multiplicity of paralogous NuRD genes would appear to permit a variety of subunit configurations, which may explain in part how NuRD functions can be fine-tuned to regulate disparate biological processes (discussed further below).

Many NuRD subunits possess protein domains that recognize specific chromatin motifs [14]. For instance, CHD3/4/5 each contain two sequential plant homeodomains (PHDs) that bind histone H3 with particular affinity for methyl (H3K9me3) and acetyl (H3K9ac) modifications [15,16,17,18,19,20]. These modifications are enriched at gene regulatory elements—both active and inactive. Conversely, PHD/nucleosome interactions are inhibited by H3K4 trimethylation, which is associated with active promoters. Research suggests that in CHD4, the tandem PHD fingers engage concomitantly with both histone H3 tails within a single nucleosome [17,18,19,21]. While chromodomains often bind to modified histone marks, *Drosophila* Mi-2 chromodomains did not exhibit such a preference [22]. The tandem chromodomains within CHD3/4/5 may instead be important for reinforcing ATPase/nucleosome interactions [23]. GATAD2A/B and RBBP4/7 subunits have also been shown to bind histones strongly in vitro [24]. GATAD2A/B and MTA1/2/3 additionally contain a single GATA-type zinc finger domain, although their affinity for DNA binding has not been well-characterized [25]. MBD3 possesses a methyl-CpG-binding domain (MBD). However, unlike other MBD proteins, MBD3 does not exhibit a preference for 5-methylcytosine in vitro [26], but instead appears to prefer 5-hydroxymethylcytosine [27]. Accordingly, both NuRD and MBD3 recruitment to the genome partially depends on DNA methylation in embryonic stem cells. On the other hand, *Drosophila* NuRD complexes appear to function similarly to their vertebrate counterparts despite the near absence of DNA methylation in flies. While this large complement of chromatin recognition modules can potentially endow NuRD with a complex valency for different chromatin states, a constellation of transcription factors have also been shown to play a role in recruiting NuRD to its targets (see below).

Genomic technologies have provided a definitive view of how chromatin states relate to NuRD occupancy. As with other chromatin remodeling complexes, NuRD is enriched at many transcriptional start sites and accessible regulatory elements [28,29]. However, NuRD complexes are also functionally associated with gene repression. Indeed, at the cell biological level, NuRD proteins are often enriched within pericentromeric heterochromatin [30,31]. Moreover, there is an intimate linkage between NuRD and Polycomb repressor complexes (PRCs) at subsets of genomic locations [28,32]. Accordingly, biochemical and functional interactions between these complexes have been repeatedly observed in neural cells (see below) [33,34,35,36].

## 3. Neurodevelopment

In humans, brain development takes many years, and includes many distinct phases and processes. Beginning at gastrulation, ectodermal cells are converted into neuroepithelial progenitors via neural induction. Neuroepithelial cells form the neural plate, and the neural tube thereafter. These progenitors concomitantly take on positional information along the rostral-caudal and dorsal-ventral axes, and thereby become specialized to produce lineages that subsequently generate and populate specific brain regions and circuits. This process begins at neurogenesis. Neuroepithelial cells differentiate into neural progenitor cells, which self-renew and produce neuronal daughter cells. Next, neural progenitors typically enter a gliogenic phase, where they produce astrocytes or oligodendrocytes. Neuronal and glial daughter cells collaborate to generate specific tissue architectures and circuit structures, with neurons responding to environmental cues to migrate, and to pathfind their axons and dendrites. In humans, the histogenesis of the brain is largely complete by the end of the second trimester. Thereafter, neural progenitor cells become depleted in most regions. Neurons are permanently postmitotic, and glial cells are quiescent. Cellular proliferation therefore wanes in most regions of the perinatal brain. However, neurodevelopment continues at a furious pace postnatally. Waves of synaptogenesis, pruning, and myelination refine circuits. Waves of apoptosis delete neurons with inappropriate connections. Infants learn at an astonishing rate, as the brain goes through critical periods for sensory experience, language, and motor tasks. A comprehensive review of brain development goes well beyond the scope of this review, but the reader is directed to excellent general reviews on brain development [41,42,43,44], as well as to reviews focused on specific brain regions that we cite in subsequent sections.

With ~80 billion neurons, ~80 billion glial cells [45], trillions of synapses, and a myriad of distinct neuroanatomical structures, it seems impossible that the development of something as complex as the human brain could ever be robust. Indeed, neurodevelopmental disorders can be traced to vulnerabilities in virtually every process sketched out in the above paragraph. Moreover, the central nervous system depends on a variety of extrinsic inputs and supports. Research suggests that neurodevelopmental disorders can also be traced to dysfunction originating outside of the central nervous system, including from the peripheral nervous system, vasculature, and maternal insults [46,47,48,49]. Importantly, evidence suggests that numerous mechanisms contribute to neurodevelopmental disorders, and that brain dysfunction does not universally map to a single neuroanatomical structure or circuit.

## 4. NuRD Complexes and Neurodevelopmental Disorders

Mutations in chromatin remodelers are prominently linked to intellectual disability (ID) and autism spectrum disorder (ASD) [50,51,52]. ID and ASD are related conditions, both in terms of etiology and phenotype, and they are frequently co-diagnosed [53,54]. The current treatment and management of these disorders is limited and often requires lifelong attention. For these reasons, ID and ASD represent an increasingly significant human and economic burden.

IDs are characterized by a generalized impairment of mental function and deficits in social and practical adaptive behaviors, which undermine the independence of afflicted individuals. Common examples include Down syndrome and Fragile X syndrome, but also conditions with environmental etiology such as fetal alcohol syndrome. The estimated prevalence of ID is approximately 1% across the population [55]. ASD affects approximately one in every ~70 children, and as many as 1 in 42 boys [56,57], and has been increasing in prevalence. Prototypical symptoms include deficits in language and social behavior, learning disability, and repetitive behaviors.

Mutations in NuRD complex genes are associated with both ID and ASD (Table 1). *CHD3* mutations lead to Snijders Blok-Campeau syndrome. *CHD4* mutations lead to Sifrim-Hitz-Weiss syndrome, while *GATAD2B* mutations lead to GATAD2B-associated neurodevelopmental disorder. Each disorder is rare, with only ~30 to 50 individuals diagnosed to date in each case. These syndromes are designated as “NuRDopathies” due to their overall similarity (reviewed in [58,59]). They are also designated as overgrowth and intellectual disability (OGID) syndromes, as afflicted individuals typically present with ID and macrocephaly [60,61,62,63,64,65]. The neurological symptoms and brain malformations associated with NuRDopathies overlap considerably, whereas the reported phenotypes in other organ systems are more divergent [59].

De novo mutations in *CHD3* and *CHD4* are clustered within the SNF2 ATPase domains and appear to structurally or functionally undermine their enzymatic functions [21,61,63,64]. Structural models of NuRD indicate that GATAD2B is critical for tethering CHD proteins to the NuRD complex [72,73], and many *GATAD2B*-associated mutations disrupt or truncate the CR1 or CR2 domains that are thought to, respectively, bind MBD3 or CHD proteins [67]. As *CHD3/4* and *GATAD2B* mutations typically undermine the chromatin remodeling module within the resultant NuRD complex, this suggests that developmental defects might be driven by the persistence of functionally abrogated complexes that might compete with wild-type NuRD for genomic occupancy.

De novo mutations in *MBD3* have additionally been observed in ASD patients, although it remains unclear whether these mutations are causative [68]. Interestingly, cerebral overgrowth is observed in many ASD patients [74,75,76], raising the possibility that the NuRD complex might regulate processes that are central to ASD etiology.

## 5. NuRD Complexes and Neural Progenitor Proliferation

NuRD function is essential for the development of the neocortex (cerebral cortex). Conditional deletion of either *Mbd3* or *Chd4* during nervous system development leads to perinatal lethality [13,77]. In both of these mutant models, neural progenitors under-proliferate, leading to neocortical hypoplasia [13,77], but the exact mechanism responsible remains to be clarified. In cell lines, CHD4 has been shown to participate in the DNA damage response [78,79,80], and CHD4 deficiency has been repeatedly shown to trigger to ATM-dependent intra-S-phase checkpoint activation [31,79,80]. The reported linkages between NuRD, heterochromatin structure, DNA damage response, and mitotic checkpoints could potentially explain the cortical hypoplasia observed in conditional mutant mice. Accordingly, elevated cell death was observed in the *Chd4* conditional knockout neocortex [13]. However, it remains much more challenging to explain why human clinical mutations in NuRD genes are instead associated with neocortical overgrowth. Brain overgrowth typically arises due to progenitor pool expansion, implying misregulation of self-renewal vs. differentiation. In mice, elevated neuronal differentiation was observed after knockdown or knockout of *Mbd3*, suggesting that gene misregulation might underlie progenitor depletion [77,81]. The contrasting phenotypes associated with mouse versus human mutations might be related to differences in genetics (conditional knockout vs. haploinsufficiency/dominant negative), or perhaps reflect evolutionary differences. In the future, it will be important to understand the mechanistic basis for these opposite effects.

## 6. NuRD Complexes and Developmental Timing

Brain function is predicated on the generation of a huge array of specialized neural cell types with different electrophysiology, morphology, and gene expression profiles. To generate neurons and glia in the correct sequences, progenitors must progressively change their developmental potential. Different phases of developmental potential are conceptualized as ‘competence states’, within which a progenitor has the potential to generate given cell-types in response to cell-intrinsic or -extrinsic cues [82,83]. Importantly, transitions in progenitor competence are well-known to be regulated by a variety of epigenetic mechanisms [84,85,86].

**Figure 3:**
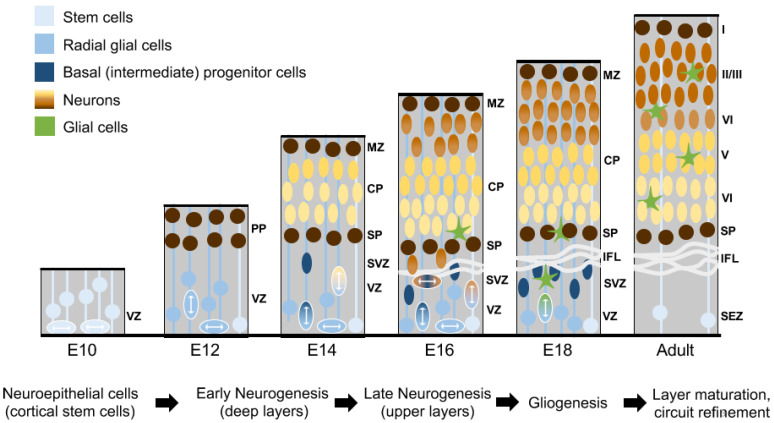
Development of the murine neocortex. At E10, neuroepithelial cells (white) in the ventricular zone (VZ) proliferate to expand the progenitor pool. By E12, neuroepithelial progenitors differentiate into radial glial cells (light blue). Radial glia begin to undergo self-renewing asymmetric divisions, producing neuronal precursors that contribute to the preplate (PP; dark brown). By E14, the preplate has split into the marginal zone (MZ) and subplate (SP). Subsequent waves of neurons migrate radially along basal processes of radial glia, and take up positions in the cortical plate (CP), settling in an inside out fashion: deep layers (yellow) are generated first and upper layers (light brown) begin to be generated later. By E14, some radial glial cells give rise to intermediate or basal progenitor cells, which in turn, are thought to divide in the subventricular zone (SVZ) to give rise to pairs of daughter neurons (shown in dark blue). Radial glial cells transition from generating neurons to generating glial cells at around E16 (green). Gliogenesis, layer maturation, axonogenesis, dendritogenesis, and circuit refinement continues to unfold postnatally, ultimately yielding the mature six-layered neocortex. IFL: inner fiber layer, SEZ: subependymal zone. I–VI: neural layer I–IV.

In vertebrates, the most thoroughly studied competence transition is the switch from neurogenesis to gliogenesis. Throughout the central nervous system, progenitors first generate neurons—often in stereotyped sequences. Most progenitors then irreversibly switch their output to produce glial cells before ceasing division [87]. During neocortical development (Figure 3), leading-edge single-cell approaches have provided an unparalleled insight into the molecular events that underlie competence transitions in vivo. These techniques have demonstrated that during the transition from neurogenesis to gliogenesis, progenitors undergo dramatic shifts in gene expression [76,88,89,90], which are accompanied by extensive chromatin remodeling [91,92].

During neocortical development, gliogenesis is timed to perinatal and postnatal stages of mouse development. Heterochromatic determinants, including DNA methylation and Polycomb, play a key role in this process [93,94]. Notably, Tsuboi et al. demonstrated that the NuRD complex cooperates with Polycomb to prevent premature neocortical gliogenesis [36]. Over developmental time, Tsuboi et al. showed that Mbd3 was increasingly recruited to the locus of *Neurog1* (*Neurogenin, Ngn1*). *Neurog1* encodes a basic helix–loop–helix transcription factor that promotes neuronal determination [95], although its role in the neocortex is nuanced [96]. *Mbd3* was in turn required for the timed recruitment of Polycomb to *Neurog1* at the onset of gliogenesis, which is concomitant with promoter deacetylation [93]. Accordingly, conditional deletion of *Mbd3* from neocortical progenitors led to prolonged and upregulated expression of the basic helix–loop–helix transcription factors *Nhlh1*, *Nhlh2*, *Neurod1*, and *Neurod2*, which are induced by Neurogenins and directly repressed by Mbd3 itself [77,97].

Interestingly, whereas Tsuboi et al. found that *Mbd3* conditional ablation led to prolonged neurogenesis during the gliogenic developmental window, Sparmann et al. found that *Chd4* knockdown led to premature gliogenesis [35]. Notably, Sparmann et al. discovered that Chd4 protein interacted with the Polycomb subunit Ezh2 in neocortical progenitors. While Rbbp4/7 were co-purified in the Chd4/Ezh2 complex, other NuRD proteins were not observed. Moreover, RNAi knockdown of *Mbd3* failed to phenocopy *Chd4* knockdown. Sparmann et al. concluded that Chd4 likely functioned independently of NuRD to suppress premature gliogenesis. Interestingly, a novel complex called ChAHP (CHD4, ADNP, HP1) has recently been described [98], which includes ADNP, a homeodomain transcription factor with prominent linkage to ASD [99]. These observations raise the possibility that CHD4 might regulate neurodevelopment independently of NuRD. On the other hand, Knock et al. found that in *Mbd3* conditional mutants, a subset of glial genes was prematurely upregulated. Taken together, these studies suggest that NuRD acts to reinforce the temporal competence state of progenitors, suppressing both premature gliogenesis during early phases of neocortical development and suppressing aberrant neurogenesis during late stages.

The above studies demonstrate considerable functional integration between NuRD and polycomb proteins. Accordingly, neocortical Polycomb mutants exhibit accelerated temporal development and premature gliogenesis [90,100]. Moreover, de novo mutations in human polycomb genes have been linked to OGID syndromes and ASD [101,102,103]. Likewise, conditional *Hdac1* and *Hdac2* double mutants resemble *Chd4* or *Mbd3* conditional knockout phenotypes in many respects, including reductions in brain size, reduced progenitor proliferation, and impaired neuronal migration [104]. Mechanistically, these linkages can be explained by the demonstrated ability of NuRD-associated HDACs to recruit polycomb to target genes [32]. However, as both polycomb and HDAC can function independently of NuRD, it remains to be formally determined whether these chromation modifiers regulate neurodevelopment through common or divergent pathways.

Additional support linking NuRD to developmental timing has been uncovered in the developing retina. Like the neocortex, the retina possesses multipotent neural progenitors that undergo competence transitions to generate neurons and glia with a stereotyped birth order [105,106]. To understand the mechanisms underlying developmental timing in the retina, we examined a zinc finger transcription factor called Casz1. We previously showed that murine Casz1 is dynamically expressed during retinal development and alters the output of retinal progenitors [107]. To understand how Casz1 functions mechanistically, we and others performed proteomics and found that Casz1 interacts with the NuRD complex [34,108]. In multipotent retinal progenitors, the interaction between Casz1 and NuRD regulated the production of neurons versus glia. When either Casz1 or the NuRD complex was abrogated, the production of earlier-born rod photoreceptors was decreased, while later-born Müller glia were concomitantly increased [34]. This effect required HDAC activity and was phenocopied by overexpressing a fragment of GATAD2A that functions as a dominant negative to block the association of Chd4 protein with NuRD [72]. Suppression of gliogenesis was also phenocopied by Polycomb loss-of-function. Although we did not formally demonstrate that NuRD controls Polycomb recruitment, Polycomb proteins were observed to associate with Casz1 [33,34], suggesting a physical linkage between these protein complexes. We propose that Casz1 suppresses gliogenesis by recruiting the NuRD complex to target genes, leading to histone deacetylation and Polycomb occupancy. An important future aspect of this work should examine the genomic changes that underlie these effects.

In the retina, interactions between NuRD and temporal transcription factors such as Casz1 may additionally explain how the NuRD complex can be redeployed at different developmental stages to control fate decisions. *Casz1* is the vertebrate orthologue of the *Drosophila* temporal transcription factor *castor*. In *Drosophila* ventral nerve cord neuroblasts, *castor* sits downstream of a cascade of sequentially expressed temporal transcription factors including the zinc finger transcription factor *hunchback* [82]. The vertebrate orthologue of hunchback is *Ikzf1* (*Ikaros*), which analogously acts upstream of Casz1 to regulate early retinal competence [109], suggesting that the fly temporal cascade is at least partly conserved in vertebrate neural lineages. Interestingly, as with Casz1, Ikzf1 is well known to interact with NuRD [110,111,112,113]. Indeed, *Drosophila* Mi-2 was first purified from a complex that included hunchback and Polycomb proteins [114]. During retinal development, NuRD might first be recruited to Ikzf1 target genes, and later shifted to Casz1 targets to orchestrate temporal development. *CASZ1* has recently been linked to ASD [2], and so it will be important to determine whether it regulates neurodevelopment elsewhere in the brain.

## 7. NuRD Complexes and Neurogenesis

Precocious gliogenesis was not observed in *Mbd3* conditional mutant neocortices. However, during early phases of neocortical development, the Gotoh lab observed that Polycomb is required to maintain the birth order of neocortical neurons [115]. Similarly, Knock et al. reported that the identities of neocortical neuronal subtypes became blurred in *Mbd3* conditional mutants, with markers of early-born and late-born neurons inappropriately coexpressed [77]. Finally, the Tole laboratory demonstrated that the NuRD complex interacts with the homeodomain transcription factor Lhx2 [116], which is a key ‘selector gene’ required for neocortical progenitor identity [117]. Muralidharan et al. showed that Lhx2 and Rbbp4 bound and suppressed *Sox11*, which encodes a transcription factor that contributes to the identity of early-born neocortical neurons [118]. Together, these studies suggest that the NuRD complex may additionally cooperate with Polycomb during early phases of neocortical development.

In addition to Casz1, and Lhx2, the NuRD complex has also been shown to physically interact with a number of additional transcription factors that are known to regulate neurogenesis and neuronal cell fate. These include transcription factors expressed in progenitors (Ikzf1/2 [119], Fezf2 [120,121,122,123,124], Myc [125,126], Sall1 [127,128], Sox2 [129,130,131]), early-born neurons (Bcl11a/b [132,133,134,135], Fezf2, Zeb2 [136,137]), and late-born neurons (Satb1/2 [138,139,140]). Importantly, formal support demonstrating that these transcription factors depend on NuRD to regulate neurodevelopment is largely lacking. Only Casz1, Lhx2, and Satb2 have thus far been shown to be necessary for NuRD recruitment, or to require NuRD for neurodevelopmental functions [10,116,139].

**Figure 4:**
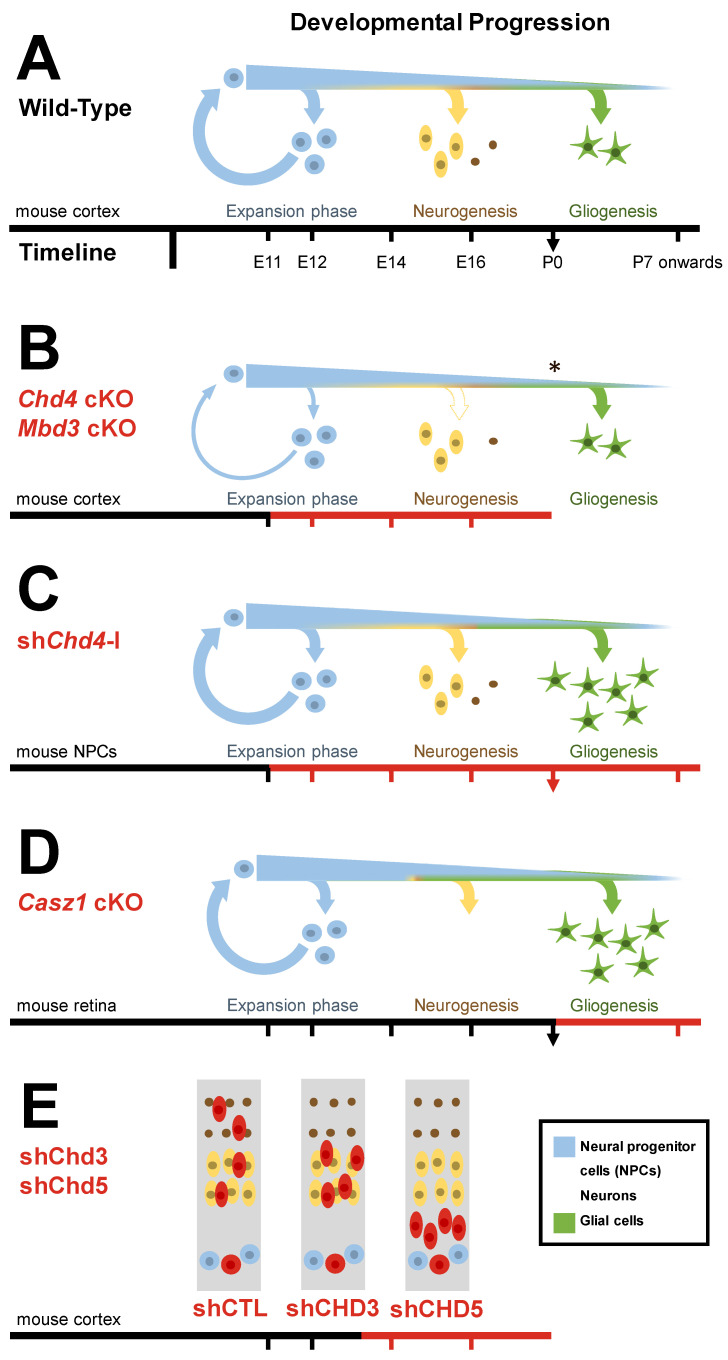
Effect of NuRD deficiency on neural lineages. Progenitors give rise to diverse neural populations in a temporally regulated manner. Selected examples illustrate how deficiencies in NuRD complex subunits impact on temporal development, potentially contributing to neurodevelopmental disorders. Red scale bars or cells indicate the genetically manipulated populations. (**A**) Wild-type. (**B**) In the *Chd4* and *Mbd3* conditional knockout neocortex, progenitors underproliferate, leading to reductions in upper layer neurons [13,77]. (**C**) Chd4 RNAi has also been linked to premature gliogenesis in neocortical cells, although this effect may be independent of NuRD [35]. (**D**) *Casz1* conditional mutant retinal progenitors overproduce glia at the expense of rod photoreceptors in a NuRD-dependent fashion [34]. (**E**) In neocortical cells, Chd3/5 RNAi disrupts neuronal migration at distinct phases [13]. Asterisk indicates lethality prior to postnatal gliogenesis.

Taken together, studies in the neocortex and retina suggest that neural progenitor cells utilize the NuRD complex to reinforce multiple temporal competence states, ensuring that cell production is properly scheduled during development. NuRD appears to be redeployed at multiple stages to repress both past and future gene expression programs (Figure 4). The repeated use of Polycomb to repress different stage-inappropriate gene expression programs via heterochromatic histone modifications might explain why nucleosome remodeling would be needed to facilitate progenitor competence transitions.

## 8. NuRD Functions in Differentiating and Mature Neurons

Understanding the roles of the NuRD complex during neuronal differentiation has been challenging, in part due to the perinatal lethality of NuRD conditional knockout mice. To circumvent these issues, Nitarska et al. used RNAi and transfection-based conditional genetics to abrogate Chd3/4/5 in a mosaic fashion, permitting functional comparison of each NuRD complex [13]. Nitarska et al. showed that while the Chd4/NuRD complex was required to sustain progenitor proliferation, Chd3 and Chd5 acted at subsequent steps of neuronal differentiation and migration. While NuRD complexes are often assumed to be ubiquitously expressed, Nitarska et al. found that Chd subunits are dynamically expressed during neocortical development, with Chd4 being most prominently expressed in progenitors, and Chd3 and Chd5 upregulating in differentiating neurons. Moreover, Chd3/4/5 were differentially recruited to target promoters, and this recruitment varied over time. Finally, abrogation of Chd3 or Chd5 could not be rescued by the complementary overexpression of alternative Chd proteins. This hints that Chd proteins exist in unique NuRD complexes with non-redundant functions, in agreement with the finding that Chd proteins are monomeric within NuRD. It also suggests that NuRD complexes can vary their functions depending on subunit composition.

While the Nitarska et al. study supports the notion that Chd4 mainly contributes to progenitor functions in the neocortex, a prominent role for Chd4 in differentiated cerebellar granule neurons has been demonstrated by the Bonni lab. As with the neocortex, the differentiation of cerebellar granule cells is accompanied by extensive chromatin remodeling [141]. In a series of landmark papers, NuRD was shown to be required for the plasticity of granule cells. ‘Neuronal plasticity’ refers to stable changes in the electrophysiological properties of neurons that are triggered by specific types of prior input. Plastic phenomena such as long-term potentiation and depression are thought to be key mechanisms that underpin learning and memory. Over the short-term, plasticity is established by signaling events that feedback to modify neurotransmission, but long-term maintenance of plasticity requires changes in gene expression [142], which are regulated in part by ‘immediate-early’ genes, including *Bdnf*, and the *Fos* and *Jun* families [143] (see below).

In the cerebellum, Chd4 is prominently expressed in differentiating granule cells. The conditional deletion of *Chd4* in postnatal granule cells led to defects in granule cell connectivity and electrophysiology [29], and thereby to behavioral deficits [39]. Yang et al. performed Chd4 ChIP-seq and found that Chd4 associated with the transcriptional start sites of nearly 10,000 genes. In conditional knockouts, Yamada et al. and Yang et al. found that *Chd4* was required to suppress promoter accessibility for a host of genes involved in synaptogenesis, as well as for immediate early genes, including *Fos*, *Jun*, and *Arc* members [29,39]. Genome-wide alterations in promoter accessibility were relatively subtle but altered promoter remodeling led to a more marked increase in the accessibility of a subset of genes (~200), correlating with increases in the active chromatin marks H3K9/14ac, H3K27ac, and H3K4me3, and transcriptional activation. Yamada et al. recapitulated these findings on a subset of genes via RNAi targeting *Gatad2a* and *Hdac1*, confirming that Chd4 functions via NuRD. 

Intriguingly, Yang et al. also found a requirement for *Chd4* to regulate variant histone deposition at target gene promoters. Whereas the expression of canonical histones is restricted to S-phase, variant histones such as H2AX, H2AZ, and H3.3 can be transcribed throughout the cell cycle [144]. Since neurons are permanently postmitotic, variant histones consequently build up over time—particularly at sites with ongoing nucleosome remodeling [145]. Yang et al. performed ChIP-seq for the variant histone H2AZ and found that *Chd4* was required for its deposition at the transcriptional start sites of immediate early genes. In the absence of *Chd4*, a lack of H2AZ deposition abrogated the repression of these genes, leading to elevated immediate early gene expression in the absence of neuronal activity [39]. Consequently, neurons exhibited defects in neurite morphology and synapse number, leading to exaggerated activity levels during motor tasks, and the impairment of motor learning. 

Interestingly, in the neocortex, immediate early genes are similarly misregulated in the absence of *Satb1*, leading to defects in the synaptic density of mutant neurons [146]. Moreover, hippocampal learning has been shown to be analogously undermined by age-related downregulation (or artificial abrogation) of Rbbp4 [147,148], suggesting that the NuRD complex might play similar roles elsewhere in the nervous system. However, as Rbbp4 and Satb1 may participate in other protein complexes, it remains to be formally established that NuRD truly underlies these effects. 

While *Chd4* remodels transcription start sites, it was also found to bind to many distal regulatory elements. Focusing on enhancers, Goodman et al. found that *Chd4* was required to restrict accessibility at enhancer elements [38]. Elevated accessibility led to increased recruitment of the cohesin complex to cognate enhancers, which altered higher-order genome organization. In general, the compartmentalization of topologically associated domains was not dramatically altered, although some domains became reassigned in *Chd4* conditional mutants. However, looping interactions within domains were markedly strengthened, elevating interactions between enhancers and promoters. Interestingly, while *Chd4* mutation leads to similar increases in accessibility at enhancers and promoters, H2AZ deposition was only reduced at promoters, whereas Ctcf recruitment was only elevated at enhancers. 

Importantly, Goodman et al. provide strong support for the notion that Chd4 suppresses cohesin binding and thereby genome looping. However, it remains to be determined whether Chd4 performs these activities via NuRD. Indeed, the recently described ChAHP complex, containing Chd4 and Adnp, was similarly shown to suppress cohesin recruitment to cryptic Ctcf sites found within repetitive elements [149]. Nonetheless, the finding that Chd4 is required to suppress accessibility at enhancer and promoter elements agrees very well with genomic work from non-neural model systems [28,113,150,151].

## 9. Future Perspectives

Human Clinical Genetics has identified a large number of genes that can contribute to ASD and ID. Despite this fact, it remains unclear how these genes lead to neurodevelopmental disorders. For example, a number of different mechanisms have been proposed to explain ASD pathogenesis, including imbalances in brain excitation versus inhibition, defective neural connectivity, and defects in neocortical neurogenesis [152,153,154,155]. Landmark research by a number of groups has provided important insight into how NuRD functions contribute to neurodevelopment.

Work by the Bonni lab in cerebellar granule cells has demonstrated that NuRD abrogation undermines neuronal plasticity, synaptogenesis, and thereby behavior. These studies have elucidated mechanisms that can directly explain the behavioral deficits associated with NuRD gene mutations. However, these studies cannot explain how NuRD mutations might lead to brain overgrowth. In earlier stages of neurodevelopment, a number of groups, including the Gotoh, Hendrich, and Riccio labs, have demonstrated that the NuRD complex regulates progenitor proliferation and temporal competence states. NuRD also regulates the migration of neocortical neurons. Going forward, the challenge will be to identify and understand the relative contribution of these processes to neurodevelopmental disorders—in progenitors, neurons, and different brain regions.

Another question that will be critical to address in the future is to understand the mechanistic basis underlying NuRD-dependent gene regulation in neural cells. In embryonic stem cells, Bornelöv and colleagues performed a high-resolution time-course analysis, revealing that NuRD occupies almost all accessible genomic regions [28]. Indeed, they found that Chd4 occupied many of these regions even in the absence of Mbd3/NuRD. NuRD recruitment to accessible sites rapidly remodeled the flanking nucleosomes and locally reduced accessibility at transcription start sites and enhancers, but interestingly, not at Ctcf sites. Shifts in nucleosome position reduced the ability of transcription factors and co-factors to be recruited to these regulatory elements. Consequently, NuRD remodeled chromatin and altered gene expression in as little as 30 min, but the expression levels of most genes were only subtly affected by NuRD activity.

The unprecedented spatial and temporal resolution of the Bornelöv study underscores the ability of NuRD to globally regulate accessible elements throughout the genome. However, a somewhat different picture emerged when examining cooperative interactions between NuRD and transcription factors. Studying the zinc finger transcription factor Ikzf1, Liang et al. found that Ikzf1 recruited NuRD to target gene promoters, leading to dramatic abrogation of gene transcription within as little as 12 min [151]. Transcriptional silencing was associated with loss of accessibility and eviction of RNA polymerase and SWI/SNF chromatin remodeling complexes. Moreover, within 24 h, target genes were repositioned into compacted, γ-satellite rich heterochromatin in a CHD4-dependent manner. These dramatic effects on target gene expression suggest that while NuRD can affect accessibility on a genome-wide basis, transcription factors might be important for facilitating more decisive decommissioning functions at particular loci. This might explain why a chromatin remodeling complex that can autonomously occupy most accessible sites throughout the genome would nonetheless depend on transcription factor interactions to regulate subsets of genes.

Research by the Bonni lab in the developing cerebellum has provided the most detailed view of how NuRD controls the genome in neural cells. Similarly to the work by Bornelöv et al., they demonstrated that NuRD has widespread recruitment to regulatory elements. Like Liang et al., they found that a subset of target genes were completely decommissioned by NuRD [29,39]. Clearly, transcription factor-dependent and -independent roles for NuRD are likely to affect neurodevelopment, and additional work will be required to assess their relative importance. Conversely, NuRD activity also remodels transcription factor occupancy [28], and it will be important to assess to what degree this activity underlies NuRD functions. Likewise, the changing landscape of NuRD paralog expression described by the Riccio lab during brain ontogeny will need to be taken into account in future work.

Finally, a critical missing piece of the puzzle is to understand how mutations associated with neurodevelopmental disorders alter NuRD complex composition and function. As the mutations associated with NuRDopathies are usually heterozygous de novo point mutations, neurodevelopmental pathogenesis may be driven by haploinsufficiency. Since NuRD abrogation affects global genome accessibility, subtle changes in complex levels could easily lead to widespread transcriptome misregulation. Alternatively, mutant proteins might incorporate into complexes, which would consequently be functionally defective. Defective complexes might compete with wild-type NuRD for genome occupancy in a dominant negative fashion. The large number of chromatin interaction modules present in NuRD subunits might also play a role in neurodevelopmental defects. Mutations that disrupt chromatin recognition or affect transcription factor interactions might lead to reduced or aberrant recruitment of NuRD to the genome. Distinguishing between these possibilities will be of critical importance for uncovering how NuRD function and dysfunction underlies neurodevelopmental disorders.

## Figures and Tables

**Table 1:** NuRD complex genes and neurodevelopmental disorders. Depicted genes are confirmed to interact with NuRD for their neurodevelopmental functions.

Gene	Mutations	Neurodevelopmental Disorders	References
*CHD3*	*de novo* (LOF)	ASD, ID, speech delay (Snijders Blok-Campeau syndrome)	[61,66]
*CHD4*	*de novo* (LOF)	ID, macrocephaly, speech delay (Sifrim-Hitz-Weiss syndrome)	[60,64]
*GATAD2B*	*de novo* (LOF)	ID, macrocephaly, epilepsy, speech delay (GATAD2B-Associated Neurodevelopmental Disorder)	[62,65,67]
*MBD3*	*de novo*	ASD	[68]
*CASZ1*	*de novo*	ASD, ID	[2,69]
*SATB2*	*de novo*	ASD, ID, epilepsy, developmental delay (SATB2-associated syndrome, Glass syndrome)	[70,71]

## Data Availability

Not applicable.

## References

[1] Niemi M.E.K., Martin H.C., Rice D.L., Gallone G., Gordon S., Kelemen M., McAloney K., McRae J., Radford E.J., Yu S. (2018). Common genetic variants contribute to risk of rare severe neurodevelopmental disorders. Nature.

[2] Wang T., Hoekzema K., Vecchio D., Wu H., Sulovari A., Coe B.P., Gillentine M.A., Wilfert A.B., Perez-Jurado L.A., Kvarnung M. (2020). Large-scale targeted sequencing identifies risk genes for neurodevelopmental disorders. Nat. Commun..

[3] Wilfert A.B., Sulovari A., Turner T.N., Coe B.P., Eichler E.E. (2017). Recurrent de novo mutations in neurodevelopmental disorders: Properties and clinical implications. Genome Med..

[4] Wright C.F., Fitzgerald T.W., Jones W.D., Clayton S., McRae J.F., van Kogelenberg M., King D.A., Ambridge K., Barrett D.M., Bayzetinova T. (2015). Genetic diagnosis of developmental disorders in the DDD study: A scalable analysis of genome-wide research data. Lancet.

[5] Wade P.A., Gegonne A., Jones P.L., Ballestar E., Aubry F., Wolffe A.P. (1999). Mi-2 complex couples DNA methylation to chromatin remodelling and histone deacetylation. Nat. Genet..

[6] Xue Y., Wong J., Moreno G.T., Young M.K., Cote J., Wang W. (1998). NURD, a novel complex with both ATP-dependent chromatin-remodeling and histone deacetylase activities. Mol. Cell.

[7] Zhang Y., LeRoy G., Seelig H.P., Lane W.S., Reinberg D. (1998). The dermatomyositis-specific autoantigen Mi2 is a component of a complex containing histone deacetylase and nucleosome remodeling activities. Cell.

[8] Low J.K.K., Silva A.P.G., Tabar M.S., Torrado M., Webb S.R., Parker B.L., Sana M., Smits C., Schmidberger J.W., Brillault L. (2020). The Nucleosome Remodeling and Deacetylase Complex Has an Asymmetric, Dynamic, and Modular Architecture. Cell Rep..

[9] Millard C.J., Fairall L., Ragan T.J., Savva C.G., Schwabe J.W.R. (2020). The topology of chromatin-binding domains in the NuRD deacetylase complex. Nucleic Acids Res..

[10] Millard C.J., Varma N., Saleh A., Morris K., Watson P.J., Bottrill A.R., Fairall L., Smith C.J., Schwabe J.W. (2016). The structure of the core NuRD repression complex provides insights into its interaction with chromatin. eLife.

[11] Zhang W., Aubert A., de Segura J.M.G., Karuppasamy M., Basu S., Murthy A.S., Diamante A., Drury T.A., Balmer J., Cramard J. (2016). The Nucleosome Remodeling and Deacetylase Complex NuRD Is Built from Preformed Catalytically Active Sub-modules. J. Mol. Biol..

[12] Hoffmeister H., Fuchs A., Erdel F., Pinz S., Grobner-Ferreira R., Bruckmann A., Deutzmann R., Schwartz U., Maldonado R., Huber C. (2017). CHD3 and CHD4 form distinct NuRD complexes with different yet overlapping functionality. Nucleic Acids Res..

[13] Nitarska J., Smith J.G., Sherlock W.T., Hillege M.M., Nott A., Barshop W.D., Vashisht A.A., Wohlschlegel J.A., Mitter R., Riccio A. (2016). A Functional Switch of NuRD Chromatin Remodeling Complex Subunits Regulates Mouse Cortical Development. Cell Rep..

[14] Allen H.F., Wade P.A., Kutateladze T.G. (2013). The NuRD architecture. Cell Mol. Life Sci..

[15] Hu Y., Liu D., Zhong X., Zhang C., Zhang Q., Zhou D.X. (2012). CHD3 protein recognizes and regulates methylated histone H3 lysines 4 and 27 over a subset of targets in the rice genome. Proc. Natl. Acad. Sci. USA.

[16] Jain K., Fraser C.S., Marunde M.R., Parker M.M., Sagum C., Burg J.M., Hall N., Popova I.K., Rodriguez K.L., Vaidya A. (2020). Characterization of the plant homeodomain (PHD) reader family for their histone tail interactions. Epigenet. Chromatin.

[17] Mansfield R.E., Musselman C.A., Kwan A.H., Oliver S.S., Garske A.L., Davrazou F., Denu J.M., Kutateladze T.G., Mackay J.P. (2011). Plant homeodomain (PHD) fingers of CHD4 are histone H3-binding modules with preference for unmodified H3K4 and methylated H3K9. J. Biol. Chem..

[18] Musselman C.A., Mansfield R.E., Garske A.L., Davrazou F., Kwan A.H., Oliver S.S., O’Leary H., Denu J.M., Mackay J.P., Kutateladze T.G. (2009). Binding of the CHD4 PHD2 finger to histone H3 is modulated by covalent modifications. Biochem. J..

[19] Musselman C.A., Ramirez J., Sims J.K., Mansfield R.E., Oliver S.S., Denu J.M., Mackay J.P., Wade P.A., Hagman J., Kutateladze T.G. (2012). Bivalent recognition of nucleosomes by the tandem PHD fingers of the CHD4 ATPase is required for CHD4-mediated repression. Proc. Natl. Acad. Sci. USA.

[20] Tencer A.H., Cox K.L., Di L., Bridgers J.B., Lyu J., Wang X., Sims J.K., Weaver T.M., Allen H.F., Zhang Y. (2017). Covalent Modifications of Histone H3K9 Promote Binding of CHD3. Cell Rep..

[21] Farnung L., Ochmann M., Cramer P. (2020). Nucleosome-CHD4 chromatin remodeler structure maps human disease mutations. eLife.

[22] Bouazoune K., Mitterweger A., Langst G., Imhof A., Akhtar A., Becker P.B., Brehm A. (2002). The dMi-2 chromodomains are DNA binding modules important for ATP-dependent nucleosome mobilization. EMBO J..

[23] Watson A.A., Mahajan P., Mertens H.D., Deery M.J., Zhang W., Pham P., Du X., Bartke T., Zhang W., Edlich C. (2012). The PHD and chromo domains regulate the ATPase activity of the human chromatin remodeler CHD4. J. Mol. Biol..

[24] Zhang W., Tyl M., Ward R., Sobott F., Maman J., Murthy A.S., Watson A.A., Fedorov O., Bowman A., Owen-Hughes T. (2013). Structural plasticity of histones H3-H4 facilitates their allosteric exchange between RbAp48 and ASF1. Nat. Struct. Mol. Biol..

[25] Feng Q., Cao R., Xia L., Erdjument-Bromage H., Tempst P., Zhang Y. (2002). Identification and functional characterization of the p66/p68 components of the MeCP1 complex. Mol. Cell. Biol..

[26] Hendrich B., Bird A. (1998). Identification and characterization of a family of mammalian methyl-CpG binding proteins. Mol. Cell. Biol..

[27] Hainer S.J., McCannell K.N., Yu J., Ee L.S., Zhu L.J., Rando O.J., Fazzio T.G. (2016). DNA methylation directs genomic localization of Mbd2 and Mbd3 in embryonic stem cells. eLife.

[28] Bornelov S., Reynolds N., Xenophontos M., Gharbi S., Johnstone E., Floyd R., Ralser M., Signolet J., Loos R., Dietmann S. (2018). The Nucleosome Remodeling and Deacetylation Complex Modulates Chromatin Structure at Sites of Active Transcription to Fine-Tune Gene Expression. Mol. Cell.

[29] Yamada T., Yang Y., Hemberg M., Yoshida T., Cho H.Y., Murphy J.P., Fioravante D., Regehr W.G., Gygi S.P., Georgopoulos K. (2014). Promoter decommissioning by the NuRD chromatin remodeling complex triggers synaptic connectivity in the mammalian brain. Neuron.

[30] Chadwick L.H., Chadwick B.P., Jaye D.L., Wade P.A. (2009). The Mi-2/NuRD complex associates with pericentromeric heterochromatin during S phase in rapidly proliferating lymphoid cells. Chromosoma.

[31] Sims J.K., Wade P.A. (2011). Mi-2/NuRD complex function is required for normal S phase progression and assembly of pericentric heterochromatin. Mol. Biol. Cell.

[32] Reynolds N., Salmon-Divon M., Dvinge H., Hynes-Allen A., Balasooriya G., Leaford D., Behrens A., Bertone P., Hendrich B. (2012). NuRD-mediated deacetylation of H3K27 facilitates recruitment of Polycomb Repressive Complex 2 to direct gene repression. EMBO J..

[33] Mattar P., Stevanovic M., Nad I., Cayouette M. (2018). Casz1 controls higher-order nuclear organization in rod photoreceptors. Proc. Natl. Acad. Sci. USA.

[34] Mattar P., Jolicoeur C., Shah S., Cayouette M. (2021). A Casz1—NuRD complex regulates temporal identity transitions in neural progenitors. Sci. Rep..

[35] Sparmann A., Xie Y., Verhoeven E., Vermeulen M., Lancini C., Gargiulo G., Hulsman D., Mann M., Knoblich J.A., van Lohuizen M. (2013). The chromodomain helicase Chd4 is required for Polycomb-mediated inhibition of astroglial differentiation. EMBO J..

[36] Tsuboi M., Kishi Y., Yokozeki W., Koseki H., Hirabayashi Y., Gotoh Y. (2018). Ubiquitination-Independent Repression of PRC1 Targets during Neuronal Fate Restriction in the Developing Mouse Neocortex. Dev. Cell.

[37] Zhong Y., Paudel B.P., Ryan D.P., Low J.K.K., Franck C., Patel K., Bedward M.J., Torrado M., Payne R.J., van Oijen A.M. (2020). CHD4 slides nucleosomes by decoupling entry- and exit-side DNA translocation. Nat. Commun..

[38] Goodman J.V., Yamada T., Yang Y., Kong L., Wu D.Y., Zhao G., Gabel H.W., Bonni A. (2020). The chromatin remodeling enzyme Chd4 regulates genome architecture in the mouse brain. Nat. Commun..

[39] Yang Y., Yamada T., Hill K.K., Hemberg M., Reddy N.C., Cho H.Y., Guthrie A.N., Oldenborg A., Heiney S.A., Ohmae S. (2016). Chromatin remodeling inactivates activity genes and regulates neural coding. Science.

[40] Clapier C.R., Iwasa J., Cairns B.R., Peterson C.L. (2017). Mechanisms of action and regulation of ATP-dependent chromatin-remodelling complexes. Nat. Rev. Mol. Cell Biol..

[41] Adnani L., Han S., Li S., Mattar P., Schuurmans C. (2018). Mechanisms of Cortical Differentiation. Int. Rev. Cell Mol. Biol..

[42] Dehaene-Lambertz G., Spelke E.S. (2015). The Infancy of the Human Brain. Neuron.

[43] Lui J.H., Hansen D.V., Kriegstein A.R. (2011). Development and evolution of the human neocortex. Cell.

[44] Silbereis J.C., Pochareddy S., Zhu Y., Li M., Sestan N. (2016). The Cellular and Molecular Landscapes of the Developing Human Central Nervous System. Neuron.

[45] Herculano-Houzel S. (2009). The human brain in numbers: A linearly scaled-up primate brain. Front. Hum. Neurosci..

[46] Knuesel I., Chicha L., Britschgi M., Schobel S.A., Bodmer M., Hellings J.A., Toovey S., Prinssen E.P. (2014). Maternal immune activation and abnormal brain development across CNS disorders. Nat. Rev. Neurol..

[47] Orefice L.L., Mosko J.R., Morency D.T., Wells M.F., Tasnim A., Mozeika S.M., Ye M., Chirila A.M., Emanuel A.J., Rankin G. (2019). Targeting Peripheral Somatosensory Neurons to Improve Tactile-Related Phenotypes in ASD Models. Cell.

[48] Orefice L.L., Zimmerman A.L., Chirila A.M., Sleboda S.J., Head J.P., Ginty D.D. (2016). Peripheral Mechanosensory Neuron Dysfunction Underlies Tactile and Behavioral Deficits in Mouse Models of ASDs. Cell.

[49] Ouellette J., Toussay X., Comin C.H., Costa L.D.F., Ho M., Lacalle-Aurioles M., Freitas-Andrade M., Liu Q.Y., Leclerc S., Pan Y. (2020). Vascular contributions to 16p11.2 deletion autism syndrome modeled in mice. Nat. Neurosci..

[50] Bjornsson H.T. (2015). The Mendelian disorders of the epigenetic machinery. Genome Res..

[51] Goodwin L.R., Picketts D.J. (2018). The role of ISWI chromatin remodeling complexes in brain development and neurodevelopmental disorders. Mol. Cell. Neurosci..

[52] Sokpor G., Castro-Hernandez R., Rosenbusch J., Staiger J.F., Tuoc T. (2018). ATP-Dependent Chromatin Remodeling During Cortical Neurogenesis. Front. Neurosci..

[53] Casanova E.L., Sharp J.L., Chakraborty H., Sumi N.S., Casanova M.F. (2016). Genes with high penetrance for syndromic and non-syndromic autism typically function within the nucleus and regulate gene expression. Mol. Autism.

[54] Thurm A., Farmer C., Salzman E., Lord C., Bishop S. (2019). State of the Field: Differentiating Intellectual Disability From Autism Spectrum Disorder. Front. Psychiatry.

[55] Maulik P.K., Mascarenhas M.N., Mathers C.D., Dua T., Saxena S. (2011). Prevalence of intellectual disability: A meta-analysis of population-based studies. Res. Dev. Disabil..

[56] Christensen D.L., Maenner M.J., Bilder D., Constantino J.N., Daniels J., Durkin M.S., Fitzgerald R.T., Kurzius-Spencer M., Pettygrove S.D., Robinson C. (2019). Prevalence and Characteristics of Autism Spectrum Disorder Among Children Aged 4 Years—Early Autism and Developmental Disabilities Monitoring Network, Seven Sites, United States, 2010, 2012, and 2014. MMWR Surveill. Summ..

[57] Developmental Disabilities Monitoring Network Surveillance Year 2010 Principal Investigators, Centers for Disease Control (2014). Prevalence of autism spectrum disorder among children aged 8 years—Autism and developmental disabilities monitoring network, 11 sites, United States, 2010. MMWR Surveill. Summ..

[58] Mossink B., Negwer M., Schubert D., Kasri N.N. (2021). The emerging role of chromatin remodelers in neurodevelopmental disorders: A developmental perspective. Cell. Mol. Life Sci..

[59] Pierson T.M., Otero M.G., Grand K., Choi A., Graham J.M., Young J.I., Mackay J.P. (2019). The NuRD complex and macrocephaly associated neurodevelopmental disorders. Am. J. Med. Genet. C Semin. Med. Genet..

[60] Sifrim A., Hitz M.P., Wilsdon A., Breckpot J., Al Turki S.H., Thienpont B., McRae J., Fitzgerald T.W., Singh T., Swaminathan G.J. (2016). Distinct genetic architectures for syndromic and nonsyndromic congenital heart defects identified by exome sequencing. Nat. Genet..

[61] Blok L.S., Rousseau J., Twist J., Ehresmann S., Takaku M., Venselaar H., Rodan L.H., Nowak C.B., Douglas J., Swoboda K.J. (2018). CHD3 helicase domain mutations cause a neurodevelopmental syndrome with macrocephaly and impaired speech and language. Nat. Commun..

[62] Vera G., Sorlin A., Delplancq G., Lecoquierre F., Brasseur-Daudruy M., Petit F., Smol T., Ziegler A., Bonneau D., Colin E. (2020). Clinical and molecular description of 19 patients with GATAD2B-Associated Neurodevelopmental Disorder (GAND). Eur. J. Med. Genet..

[63] Weiss K., Lazar H.P., Kurolap A., Martinez A.F., Paperna T., Cohen L., Smeland M.F., Whalen S., Heide S., Keren B. (2020). The CHD4-related syndrome: A comprehensive investigation of the clinical spectrum, genotype-phenotype correlations, and molecular basis. Genet. Med..

[64] Weiss K., Terhal P.A., Cohen L., Bruccoleri M., Irving M., Martinez A.F., Rosenfeld J.A., Machol K., Yang Y., Liu P. (2016). De Novo Mutations in CHD4, an ATP-Dependent Chromatin Remodeler Gene, Cause an Intellectual Disability Syndrome with Distinctive Dysmorphisms. Am. J. Hum. Genet..

[65] Willemsen M.H., Nijhof B., Fenckova M., Nillesen W.M., Bongers E.M.H.F., Castells-Nobau A., Asztalos L., Viragh E., van Bon B.W.M., Tezel E. (2013). GATAD2B loss-of-function mutations cause a recognisable syndrome with intellectual disability and are associated with learning deficits and synaptic undergrowth in Drosophila. J. Med. Genet..

[66] Iossifov I., O’Roak B.J., Sanders S.J., Ronemus M., Krumm N., Levy D., Stessman H.A., Witherspoon K.T., Vives L., Patterson K.E. (2014). The contribution of de novo coding mutations to autism spectrum disorder. Nature.

[67] Shieh C., Jones N., Vanle B., Au M., Huang A.Y., Silva A.P.G., Lee H., Douine E.D., Otero M.G., Choi A. (2020). GATAD2B-associated neurodevelopmental disorder (GAND): Clinical and molecular insights into a NuRD-related disorder. Genet. Med..

[68] Cukier H.N., Rabionet R., Konidari I., Rayner-Evans M.Y., Baltos M.L., Wright H.H., Abramson R.K., Martin E.R., Cuccaro M.L., Pericak-Vance M.A. (2010). Novel variants identified in methyl-CpG-binding domain genes in autistic individuals. Neurogenetics.

[69] Coe B.P., Stessman H.A.F., Sulovari A., Geisheker M.R., Bakken T.E., Lake A.M., Dougherty J.D., Lein E.S., Hormozdiari F., Bernier R.A. (2019). Neurodevelopmental disease genes implicated by de novo mutation and copy number variation morbidity. Nat. Genet..

[70] Chevarin M., Duffourd Y., Barnard R.A., Moutton S., Lecoquierre F., Daoud F., Kuentz P., Cabret C., Thevenon J., Gautier E. (2020). Excess of de novo variants in genes involved in chromatin remodelling in patients with marfanoid habitus and intellectual disability. J. Med. Genet..

[71] Talkowski M.E., Rosenfeld J.A., Blumenthal I., Pillalamarri V., Chiang C., Heilbut A., Ernst C., Hanscom C., Rossin E., Lindgren A.M. (2012). Sequencing chromosomal abnormalities reveals neurodevelopmental loci that confer risk across diagnostic boundaries. Cell.

[72] Sher F., Hossain M., Seruggia D., Schoonenberg V.A.C., Yao Q., Cifani P., Dassama L.M.K., Cole M.A., Ren C., Vinjamur D.S. (2019). Rational targeting of a NuRD subcomplex guided by comprehensive in situ mutagenesis. Nat. Genet..

[73] Torrado M., Low J.K.K., Silva A.P.G., Schmidberger J.W., Sana M., Tabar M.S., Isilak M.E., Winning C.S., Kwong C., Bedward M.J. (2017). Refinement of the subunit interaction network within the nucleosome remodelling and deacetylase (NuRD) complex. FEBS J..

[74] Courchesne E. (2002). Abnormal early brain development in autism. Mol. Psychiatry.

[75] Courchesne E., Campbell K., Solso S. (2011). Brain growth across the life span in autism: Age-specific changes in anatomical pathology. Brain Res..

[76] Lee J.K., Andrews D.S., Ozonoff S., Solomon M., Rogers S., Amaral D.G., Nordahl C.W. (2020). Longitudinal Evaluation of Cerebral Growth Across Childhood in Boys and Girls With Autism Spectrum Disorder. Biol. Psychiatry.

[77] Knock E., Pereira J., Lombard P.D., Dimond A., Leaford D., Livesey F.J., Hendrich B. (2015). The methyl binding domain 3/nucleosome remodelling and deacetylase complex regulates neural cell fate determination and terminal differentiation in the cerebral cortex. Neural Dev..

[78] Luijsterburg M.S., Acs K., Ackermann L., Wiegant W.W., Bekker-Jensen S., Larsen D.H., Khanna K.K., van Attikum H., Mailand N., Dantuma N.P. (2012). A new non-catalytic role for ubiquitin ligase RNF8 in unfolding higher-order chromatin structure. EMBO J..

[79] Polo S.E., Kaidi A., Baskcomb L., Galanty Y., Jackson S.P. (2010). Regulation of DNA-damage responses and cell-cycle progression by the chromatin remodelling factor CHD4. EMBO J..

[80] Smeenk G., Wiegant W.W., Vrolijk H., Solari A.P., Pastink A., van Attikum H. (2010). The NuRD chromatin-remodeling complex regulates signaling and repair of DNA damage. J. Cell Biol..

[81] Moon B.S., Yun H.M., Chang W.H., Steele B.H., Cai M., Choi S.H., Lu W. (2017). Smek promotes corticogenesis through regulating Mbd3’s stability and Mbd3/NuRD complex recruitment to genes associated with neurogenesis. PLoS Biol..

[82] Kohwi M., Doe C.Q. (2013). Temporal fate specification and neural progenitor competence during development. Nat. Rev. Neurosci..

[83] Livesey F.J., Cepko C.L. (2001). Vertebrate neural cell-fate determination: Lessons from the retina. Nat. Rev. Neurosci..

[84] Albert M., Huttner W.B. (2018). Epigenetic and Transcriptional Pre-patterning-An Emerging Theme in Cortical Neurogenesis. Front. Neurosci..

[85] Amberg N., Laukoter S., Hippenmeyer S. (2019). Epigenetic cues modulating the generation of cell-type diversity in the cerebral cortex. J. Neurochem..

[86] Yoon K.J., Vissers C., Ming G.L., Song H. (2018). Epigenetics and epitranscriptomics in temporal patterning of cortical neural progenitor competence. J. Cell Biol..

[87] Miller F.D., Gauthier A.S. (2007). Timing is everything: Making neurons versus glia in the developing cortex. Neuron.

[88] Loo L., Simon J.M., Xing L., McCoy E.S., Niehaus J.K., Guo J., Anton E.S., Zylka M.J. (2019). Single-cell transcriptomic analysis of mouse neocortical development. Nat. Commun..

[89] Nowakowski T.J., Bhaduri A., Pollen A.A., Alvarado B., Mostajo-Radji M.A., Di Lullo E., Haeussler M., Sandoval-Espinosa C., Liu S.J., Velmeshev D. (2017). Spatiotemporal gene expression trajectories reveal developmental hierarchies of the human cortex. Science.

[90] Telley L., Agirman G., Prados J., Amberg N., Fievre S., Oberst P., Bartolini G., Vitali I., Cadilhac C., Hippenmeyer S. (2019). Temporal patterning of apical progenitors and their daughter neurons in the developing neocortex. Science.

[91] De la Torre-Ubieta L., Stein J.L., Won H., Opland C.K., Liang D., Lu D., Geschwind D.H. (2018). The Dynamic Landscape of Open Chromatin during Human Cortical Neurogenesis. Cell.

[92] Preissl S., Fang R., Huang H., Zhao Y., Raviram R., Gorkin D.U., Zhang Y., Sos B.C., Afzal V., Dickel D.E. (2018). Single-nucleus analysis of accessible chromatin in developing mouse forebrain reveals cell-type-specific transcriptional regulation. Nat. Neurosci..

[93] Hirabayashi Y., Suzki N., Tsuboi M., Endo T.A., Toyoda T., Shinga J., Koseki H., Vidal M., Gotoh Y. (2009). Polycomb limits the neurogenic competence of neural precursor cells to promote astrogenic fate transition. Neuron.

[94] Takizawa T., Nakashima K., Namihira M., Ochiai W., Uemura A., Yanagisawa M., Fujita N., Nakao M., Taga T. (2001). DNA methylation is a critical cell-intrinsic determinant of astrocyte differentiation in the fetal brain. Dev. Cell.

[95] Ma Q., Kintner C., Anderson D.J. (1996). Identification of neurogenin, a vertebrate neuronal determination gene. Cell.

[96] Han S., Dennis D.J., Balakrishnan A., Dixit R., Britz O., Zinyk D., Touahri Y., Olender T., Brand M., Guillemot F. (2018). A non-canonical role for the proneural gene Neurog1 as a negative regulator of neocortical neurogenesis. Development.

[97] Mattar P., Langevin L.M., Markham K., Klenin N., Shivji S., Zinyk D., Schuurmans C. (2008). Basic helix-loop-helix transcription factors cooperate to specify a cortical projection neuron identity. Mol. Cell. Biol..

[98] Ostapcuk V., Mohn F., Carl S.H., Basters A., Hess D., Iesmantavicius V., Lampersberger L., Flemr M., Pandey A., Thoma N.H. (2018). Activity-dependent neuroprotective protein recruits HP1 and CHD4 to control lineage-specifying genes. Nature.

[99] Helsmoortel C., Vulto-van Silfhout A.T., Coe B.P., Vandeweyer G., Rooms L., van den Ende J., Schuurs-Hoeijmakers J.H., Marcelis C.L., Willemsen M.H., Vissers L.E. (2014). A SWI/SNF-related autism syndrome caused by de novo mutations in ADNP. Nat. Genet..

[100] Pereira J.D., Sansom S.N., Smith J., Dobenecker M.W., Tarakhovsky A., Livesey F.J. (2010). Ezh2, the histone methyltransferase of PRC2, regulates the balance between self-renewal and differentiation in the cerebral cortex. Proc. Natl. Acad. Sci. USA.

[101] Choufani S., Gibson W.T., Turinsky A.L., Chung B.H.Y., Wang T., Garg K., Vitriolo A., Cohen A.S.A., Cyrus S., Goodman S. (2020). DNA Methylation Signature for EZH2 Functionally Classifies Sequence Variants in Three PRC2 Complex Genes. Am. J. Hum. Genet..

[102] Cyrus S., Burkardt D., Weaver D.D., Gibson W.T. (2019). PRC2-complex related dysfunction in overgrowth syndromes: A review of EZH2, EED, and SUZ12 and their syndromic phenotypes. Am. J. Med. Genet. C Semin. Med. Genet..

[103] Gao Z., Lee P., Stafford J.M., von Schimmelmann M., Schaefer A., Reinberg D. (2014). An AUTS2-Polycomb complex activates gene expression in the CNS. Nature.

[104] Montgomery R.L., Hsieh J., Barbosa A.C., Richardson J.A., Olson E.N. (2009). Histone deacetylases 1 and 2 control the progression of neural precursors to neurons during brain development. Proc. Natl. Acad. Sci. USA.

[105] Bassett E.A., Wallace V.A. (2012). Cell fate determination in the vertebrate retina. Trends Neurosci..

[106] Cepko C. (2014). Intrinsically different retinal progenitor cells produce specific types of progeny. Nat. Rev. Neurosci..

[107] Mattar P., Ericson J., Blackshaw S., Cayouette M. (2015). A conserved regulatory logic controls temporal identity in mouse neural progenitors. Neuron.

[108] Liu Z., Lam N., Thiele C.J. (2015). Zinc finger transcription factor CASZ1 interacts with histones, DNA repair proteins and recruits NuRD complex to regulate gene transcription. Oncotarget.

[109] Elliott J., Jolicoeur C., Ramamurthy V., Cayouette M. (2008). Ikaros confers early temporal competence to mouse retinal progenitor cells. Neuron.

[110] Bottardi S., Mavoungou L., Pak H., Daou S., Bourgoin V., Lakehal Y.A., Affar el B., Milot E. (2014). The IKAROS interaction with a complex including chromatin remodeling and transcription elongation activities is required for hematopoiesis. PLoS Genet..

[111] Dege C., Hagman J. (2014). Mi-2/NuRD chromatin remodeling complexes regulate B and T-lymphocyte development and function. Immunol. Rev..

[112] Kim J., Sif S., Jones B., Jackson A., Koipally J., Heller E., Winandy S., Viel A., Sawyer A., Ikeda T. (1999). Ikaros DNA-binding proteins direct formation of chromatin remodeling complexes in lymphocytes. Immunity.

[113] Yoshida T., Hu Y., Zhang Z., Emmanuel A.O., Galani K., Muhire B., Snippert H.J., Williams C.J., Tolstorukov M.Y., Gounari F. (2019). Chromatin restriction by the nucleosome remodeler Mi-2beta and functional interplay with lineage-specific transcription regulators control B-cell differentiation. Genes Dev..

[114] Kehle J., Beuchle D., Treuheit S., Christen B., Kennison J.A., Bienz M., Muller J. (1998). dMi-2, a hunchback-interacting protein that functions in polycomb repression. Science.

[115] Morimoto-Suzki N., Hirabayashi Y., Tyssowski K., Shinga J., Vidal M., Koseki H., Gotoh Y. (2014). The polycomb component Ring1B regulates the timed termination of subcerebral projection neuron production during mouse neocortical development. Development.

[116] Muralidharan B., Khatri Z., Maheshwari U., Gupta R., Roy B., Pradhan S.J., Karmodiya K., Padmanabhan H., Shetty A.S., Balaji C. (2017). LHX2 Interacts with the NuRD Complex and Regulates Cortical Neuron Subtype Determinants Fezf2 and Sox11. J. Neurosci..

[117] Mangale V.S., Hirokawa K.E., Satyaki P.R., Gokulchandran N., Chikbire S., Subramanian L., Shetty A.S., Martynoga B., Paul J., Mai M.V. (2008). Lhx2 selector activity specifies cortical identity and suppresses hippocampal organizer fate. Science.

[118] Shim S., Kwan K.Y., Li M., Lefebvre V., Sestan N. (2012). Cis-regulatory control of corticospinal system development and evolution. Nature.

[119] Alsio J.M., Tarchini B., Cayouette M., Livesey F.J. (2013). Ikaros promotes early-born neuronal fates in the cerebral cortex. Proc. Natl. Acad. Sci. USA.

[120] Chen B., Schaevitz L.R., McConnell S.K. (2005). Fezl regulates the differentiation and axon targeting of layer 5 subcortical projection neurons in cerebral cortex. Proc. Natl. Acad. Sci. USA.

[121] Chen J.G., Rasin M.R., Kwan K.Y., Sestan N. (2005). Zfp312 is required for subcortical axonal projections and dendritic morphology of deep-layer pyramidal neurons of the cerebral cortex. Proc. Natl. Acad. Sci. USA.

[122] Hirata T., Suda Y., Nakao K., Narimatsu M., Hirano T., Hibi M. (2004). Zinc finger gene fez-like functions in the formation of subplate neurons and thalamocortical axons. Dev. Dyn..

[123] Molyneaux B.J., Arlotta P., Hirata T., Hibi M., Macklis J.D. (2005). Fezl is required for the birth and specification of corticospinal motor neurons. Neuron.

[124] Tomofuji Y., Takaba H., Suzuki H.I., Benlaribi R., Martinez C.D.P., Abe Y., Morishita Y., Okamura T., Taguchi A., Kodama T. (2020). Chd4 choreographs self-antigen expression for central immune tolerance. Nat. Immunol..

[125] Knoepfler P.S., Cheng P.F., Eisenman R.N. (2002). N-myc is essential during neurogenesis for the rapid expansion of progenitor cell populations and the inhibition of neuronal differentiation. Genes Dev..

[126] Rais Y., Zviran A., Geula S., Gafni O., Chomsky E., Viukov S., Mansour A.A., Caspi I., Krupalnik V., Zerbib M. (2013). Deterministic direct reprogramming of somatic cells to pluripotency. Nature.

[127] Harrison S.J., Nishinakamura R., Jones K.R., Monaghan A.P. (2012). Sall1 regulates cortical neurogenesis and laminar fate specification in mice: Implications for neural abnormalities in Townes-Brocks syndrome. Dis. Models Mech..

[128] Lauberth S.M., Rauchman M. (2006). A conserved 12-amino acid motif in Sall1 recruits the nucleosome remodeling and deacetylase corepressor complex. J. Biol. Chem..

[129] Ferri A.L.M., Cavallaro M., Braida D., Di Cristofano A., Canta A., Vezzani A., Ottolenghi S., Pandolfi P.P., Sala M., DeBiasi S. (2004). Sox2 deficiency causes neurodegeneration and impaired neurogenesis in the adult mouse brain. Development.

[130] Mallanna S.K., Ormsbee B.D., Iacovino M., Gilmore J.M., Cox J.L., Kyba M., Washburn M.P., Rizzino A. (2010). Proteomic analysis of Sox2-associated proteins during early stages of mouse embryonic stem cell differentiation identifies Sox21 as a novel regulator of stem cell fate. Stem Cells.

[131] Miyagi S., Masui S., Niwa H., Saito T., Shimazaki T., Okano H., Nishimoto M., Muramatsu M., Iwama A., Okuda A. (2008). Consequence of the loss of Sox2 in the developing brain of the mouse. FEBS Lett..

[132] Arlotta P., Molyneaux B.J., Jabaudon D., Yoshida Y., Macklis J.D. (2008). Ctip2 controls the differentiation of medium spiny neurons and the establishment of the cellular architecture of the striatum. J. Neurosci..

[133] Greig L.C., Woodworth M.B., Greppi C., Macklis J.D. (2016). Ctip1 Controls Acquisition of Sensory Area Identity and Establishment of Sensory Input Fields in the Developing Neocortex. Neuron.

[134] Sankaran V.G., Menne T.F., Xu J., Akie T.E., Lettre G., Van Handel B., Mikkola H.K.A., Hirschhorn J.N., Cantor A.B., Orkin S.H. (2008). Human fetal hemoglobin expression is regulated by the developmental stage-specific repressor BCL11A. Science.

[135] Woodworth M.B., Greig L.C., Liu K.X., Ippolito G.C., Tucker H.O., Macklis J.D. (2016). Ctip1 Regulates the Balance between Specification of Distinct Projection Neuron Subtypes in Deep Cortical Layers. Cell Rep..

[136] Seuntjens E., Nityanandam A., Miquelajauregui A., Debruyn J., Stryjewska A., Goebbels S., Nave K.A., Huylebroeck D., Tarabykin V. (2009). Sip1 regulates sequential fate decisions by feedback signaling from postmitotic neurons to progenitors. Nat. Neurosci..

[137] Verstappen G., van Grunsven L.A., Michiels C., Van de Putte T., Souopgui J., Van Damme J., Bellefroid E., Vandekerckhove J., Huylebroeck D. (2008). Atypical Mowat-Wilson patient confirms the importance of the novel association between ZFHX1B/SIP1 and NuRD corepressor complex. Hum. Mol. Genet..

[138] Alcamo E.A., Chirivella L., Dautzenberg M., Dobreva G., Farinas I., Grosschedl R., McConnell S.K. (2008). Satb2 regulates callosal projection neuron identity in the developing cerebral cortex. Neuron.

[139] Britanova O., de Juan Romero C., Cheung A., Kwan K.Y., Schwark M., Gyorgy A., Vogel T., Akopov S., Mitkovski M., Agoston D. (2008). Satb2 is a postmitotic determinant for upper-layer neuron specification in the neocortex. Neuron.

[140] Yasui D., Miyano M., Cai S., Varga-Weisz P., Kohwi-Shigematsu T. (2002). SATB1 targets chromatin remodelling to regulate genes over long distances. Nature.

[141] Frank C.L., Liu F., Wijayatunge R., Song L., Biegler M.T., Yang M.G., Vockley C.M., Safi A., Gersbach C.A., Crawford G.E. (2015). Regulation of chromatin accessibility and Zic binding at enhancers in the developing cerebellum. Nat. Neurosci..

[142] West A.E., Greenberg M.E. (2011). Neuronal activity-regulated gene transcription in synapse development and cognitive function. Cold Spring Harb. Perspect. Biol..

[143] Minatohara K., Akiyoshi M., Okuno H. (2015). Role of Immediate-Early Genes in Synaptic Plasticity and Neuronal Ensembles Underlying the Memory Trace. Front. Mol. Neurosci..

[144] Henikoff S., Smith M.M. (2015). Histone variants and epigenetics. Cold Spring Harb. Perspect. Biol..

[145] Stefanelli G., Azam A.B., Walters B.J., Brimble M.A., Gettens C.P., Bouchard-Cannon P., Cheng H.M., Davidoff A.M., Narkaj K., Day J.J. (2018). Learning and Age-Related Changes in Genome-wide H2A.Z Binding in the Mouse Hippocampus. Cell Rep..

[146] Balamotis M.A., Tamberg N., Woo Y.J., Li J., Davy B., Kohwi-Shigematsu T., Kohwi Y. (2012). Satb1 ablation alters temporal expression of immediate early genes and reduces dendritic spine density during postnatal brain development. Mol. Cell. Biol..

[147] Kosmidis S., Polyzos A., Harvey L., Youssef M., Denny C.A., Dranovsky A., Kandel E.R. (2018). RbAp48 Protein Is a Critical Component of GPR158/OCN Signaling and Ameliorates Age-Related Memory Loss. Cell Rep..

[148] Pavlopoulos E., Jones S., Kosmidis S., Close M., Kim C., Kovalerchik O., Small S.A., Kandel E.R. (2013). Molecular mechanism for age-related memory loss: The histone-binding protein RbAp48. Sci. Transl. Med..

[149] Kaaij L.J.T., Mohn F., van der Weide R.H., de Wit E., Buhler M. (2019). The ChAHP Complex Counteracts Chromatin Looping at CTCF Sites that Emerged from SINE Expansions in Mouse. Cell.

[150] Arends T., Dege C., Bortnick A., Danhorn T., Knapp J.R., Jia H., Harmacek L., Fleenor C.J., Straign D., Walton K. (2019). CHD4 is essential for transcriptional repression and lineage progression in B lymphopoiesis. Proc. Natl. Acad. Sci. USA.

[151] Liang Z., Brown K.E., Carroll T., Taylor B., Vidal I.F., Hendrich B., Rueda D., Fisher A.G., Merkenschlager M. (2017). A high-resolution map of transcriptional repression. eLife.

[152] Chao H.T., Chen H., Samaco R.C., Xue M., Chahrour M., Yoo J., Neul J.L., Gong S., Lu H.C., Heintz N. (2010). Dysfunction in GABA signalling mediates autism-like stereotypies and Rett syndrome phenotypes. Nature.

[153] Kwan K.Y. (2013). Transcriptional dysregulation of neocortical circuit assembly in ASD. Int. Rev. Neurobiol..

[154] Rubenstein J.L., Merzenich M.M. (2003). Model of autism: Increased ratio of excitation/inhibition in key neural systems. Genes Brain Behav..

[155] Walsh C.A., Morrow E.M., Rubenstein J.L. (2008). Autism and brain development. Cell.

